# Optimal Design of RF Energy-Harvesting Network: Throughput and Delay Perspective

**DOI:** 10.3390/s19010145

**Published:** 2019-01-03

**Authors:** Yun Han Bae, Jung Woo Baek

**Affiliations:** 1 Department of Mathematics Education, Sangmyung University, Seoul 03016, Korea; yhbae@smu.ac.kr; 2 Department of Industrial Engineering, Chosun University, Gwangju 61452, Korea

**Keywords:** optimal transmission probability, RF energy harvesting, packet delay, throughput

## Abstract

We consider a wireless network comprising two types of nodes: Type I node and Type II node. The Type I node has unlimited energy supply and the Type II node is powered by radio frequency (RF) energy harvesting where the RF transmissions by the Type I node are the opportunities for the Type II node to replenish its battery. The Type I node has a data queue for storing data packets generated intermittently and the Type II node has a backlogged data queue. Our objective in this paper is to investigate system throughput and packet delay in an RF energy-harvesting network. Specifically, we find the optimal transmission probabilities of the two nodes which minimize the packet delay of the Type I node while maximizing the system throughput subject to the stability condition of the data queue of the Type I node. The whole system of the two interacting nodes can be exactly modeled by a two-dimensional Markov chain. Instead of following such an approach, we resort to another approximate approach so that our optimization problem can be solved more easily and closed-form solutions are available. The accuracy of the approximate model is validated by extensive simulations.

## 1. Introduction

The widespread use of battery-powered wireless devices makes extending the lifetime of these devices more important [1]. Radio frequency (RF) energy harvesting has received much attention as a possible solution for replenishing energy without any physical connections for charging. Nodes equipped with RF energy harvesting can harvest energy from ambient RF sources (e.g., TV, radio towers, and cellular base stations) and use this energy for communication operations. Recent studies [2,3] have shown that RF energy harvesting is a feasible technique based on experimental measurements of various RF energy sources. Please refer to the recent survey paper [4] on the contemporary research in wireless networks with RF energy transfer and harvesting.

Traditional medium access control (MAC) protocols (e.g., slotted Aloha [5] and CSMA/CA [6]) have mainly been designed with the objective of optimizing system performance (e.g., system throughput and packet delay) under the assumption that there is no energy availability constraint on devices. RF energy harvesting introduces new dimensions into the wireless communication problem due to the intermittency and randomness of the available energy [7]. Thus, MAC protocols incorporating RF energy harvesting must be redesigned to optimize the system performance under energy availability constraint.

In this paper, we consider a wireless network comprising two types of nodes; Type I node and Type II node. The Type I node has unlimited energy supply and the Type II node is powered by RF energy harvesting. The RF transmissions of the Type I node are opportunities for the Type II node to harvest energy. The Type II node operates in a half-duplex mode in that it can either harvest energy from the RF transmission by the Type I node or transmit a data packet at a given instant, not both simultaneously. The Type I node has a data queue for storing data packets generated intermittently. The Type II node has an energy queue for storing energy harvested from the RF transmissions of the Type I node and has a data queue which is always saturated, i.e., full of data packets to send. A wireless channel is shared between the two nodes based on the slotted Aloha where each node uses a different transmission probability. The two nodes are interacting with each other in that the transmissions of the Type I node provide the Type II node with the opportunities for energy harvesting and in turn the transmissions of the Type II node interfere with the transmission of the Type I node.

An example scenario for the network model considered in this paper may be a WLAN coexisting with RF energy-harvesting sensor nodes. In [8,9], RF energy harvesting of sensor nodes from 2.45 GHz indoor Wi-Fi signals were investigated. Sensor nodes are deployed in an indoor environment. A WLAN access point or devices, which have unlimited energy supply, emit RF signals for data transmission on the WLAN band from which sensor nodes can harvest energy for its operation purpose. Two types of nodes that coexist in a WLAN and share the same WLAN band, compete with each other for their data transmission according to a randem access rule. The network model considered in this paper is also similar to the scenarios considered in [10,11,12]. The works [10,11] considered a slotted Aloha system consisting of one energy-harvesting node and two energy-harvesting nodes, respectively. They [10,11] derived the stability region, the set of data arrival rate vectors for which all data queues in the system are stable, of the packet queues under the energy causality constraint. The work [12] considered a slotted Aloha system consisting of two nodes where the Type I node has an unlimited energy supply and the Type II node is in the energy-limited phase in that the required energy is harvested from the RF transmissions of the Type I node, which is the same scenario assumed in this paper. They also obtained the stability region of the data packet queues. All of these works focused on only stability analysis of network queues. For a slotted Aloha system with energy harvesting, system throughput and packet delay analysis taking into account the energy causality constraint still remains an open issue. Specifically, the optimal aloha transmission probability while satisfying the throughput and packet delay constraint and the energy causality constraint has not been investigated so far.

For a slotted Aloha system with RF energy harvesting, all network queues (data and energy queues) are interacting with each other in the sense that the service process of one depends on the status of the others. This is the reason why most previous work has focused on small-sized networks [10,11,12] or approximation techniques (e.g., [13,14]) are used for analysis. From a mathematical modeling point of view, this coupled system can be modeled as a multi-dimensional Markov chain. In order to optimize this system in terms of several performance measures such as packet delay and throughput, we need to first derive the steady-state distribution of the Markov chain and then using it the system may be optimized. In doing so, matrix-analytic methods [15] may be applied for deriving the steady-state distribution. This approach is computationally efficient, but it is difficult to obtain an insight on how various system parameters affect system performance. Instead of this approach, we develop an approximate modeling scheme. The main contributions of this paper are summarized as follows:

- To analyze the coupled queueing system, we develop an approximate analytical model based on an appropriate decoupling approximation. This approximate model has a closed-form steady-state solution, which can be used to optimize the system performance efficiently.
- We derive the system throughput for the two different phases in which the Type II node may be in *the energy-limited phase* and *the energy-unlimited phase* by controlling its transmission probability. We obtain the optimal transmission probabilities to maximize the system throughput while satisfying the stability condition of the data queue of the Type I node.
- We derive the mean packet delay of the Type I node. Finally, we derive the optimal transmission probabilities to minimize the packet delay of the Type I node while maximizing the system throughput subject to the stability condition of the data queue of the Type I node.

There have been several works which focus on design and analysis of energy harvesting-aware MAC protocols for various wireless energy-harvesting networks. Mekikis et al. [16] investigated the performance of communication in dense networks with wireless energy-harvesting nodes. They [16] derived theoretical expressions for the probability of successful communication for two different communication scenarios (direct and cooperative). Mekikis et al. [17] also considered a large-scale two-way network coding-aided cooperative network where the relay nodes are equipped with electromagnetic radiation energy-harvesting capabilities. They [17] obtained theoretical expressions for the probability of successful data exchange and the network lifetime gain. For a three-node wireless powered communication system, Zhao et al. [18] achieved the maximum throughput by balancing the wireless power transfer phase and the information transfer phase under the energy causality constraint, the time duration constraint and the quality-of-service (QoS) constraint. Ibarra et al. [19] considered QoS-aware energy management scheme for body sensor nodes in wireless body area networks. They [19] proposed a power-QoS control scheme designed for body sensor nodes in wireless body area networks with human energy harvesting. Han et al. [20] modeled and analyzed a large-scale backscatter communication network using stochastic geometry where while they proposed a novel network architecture that enables device-to-device communication between passive nodes by integrating wireless power transfer and backscatter communication. Esteves et al. [21] proposed a cooperative energy-harvesting MAC protocol in wireless body area networks with energy harvesting which is an adaptive MAC in the sense that a charging period that enables the network relays to harvest the required energy can be dynamically chosen based on the available energy level of the nodes.

The Aloha protocol, as well as its variants, have attracted much attention since it was proposed by Abramson [5] because of its simplicity and decentralized nature. However, with the introduction of an energy-harvesting technique Aloha-type protocols need to be redesigned because the energy availability constraints significantly affect the system performance. Several previous studies have considered multi-access wireless networks incorporating energy harvesting. In [22], the authors investigated the optimal packet scheduling problem in a two-user multiple access communication system with RF energy harvesting. In [23], the authors considered the problem of data fusion in a wireless sensor network comprising sensors with energy harvesting, where a random-access protocol was designed by characterizing a symmetric Nash equilibrium. In [24], the focus was to redesign traditional random access protocols by investigating the interplay between the delivery probability and time efficiency in wireless sensor networks powered by energy harvesting. The studies [10,11] dealt with the stability issue for packet queues for the two-node slotted-Aloha system with RF energy harvesting. Pappas et al. [25] also determined the stability region for the interacting queues in a two-hop cooperative network based on slotted Aloha with RF energy harvesting. In [26], the authors obtained the spatial throughput in a mobile network with RF energy harvesting using a stochastic-geometry model where transmitters were distributed in a plane according to a Poisson point process. In [27], the authors derived the optimal Aloha transmission probability that maximizes the transmission capacity for a multi-user Aloha access network with RF energy harvesting by coupling the energy queue dynamics with the Aloha transmission probability. In our previous study [28], we considered a multi-user cognitive radio network based on slotted Aloha with energy harvesting. The main finding in [28] was that the maximum system throughput is insensitive to the energy queue capacity and we derived the optimal sensing and transmission probabilities that maximize the system throughput.

In [4], dynamic spectrum access in the RF-powered cognitive radio network was considered and the authors investigated the optimal channel selection policy to maximize the throughput for secondary users under the energy availability constraint. The study [12] is closely related to our work in terms of the network model considered. In [12], the authors investigated a slotted-Aloha access wireless network comprising two nodes with and without RF energy-harvesting capabilities. They investigated the effects of RF energy harvesting on the stability region for interacting queues in a slotted Aloha system. However, they only focused on the stability issue. The focus in this paper is to design the optimal transmission probabilities for optimizing the system throughput and the packet delay. This issue was not addressed in the literature mentioned so far. This paper and our previous work [13] share a common interest in mathematical modeling approach. In [13], we proposed an approximate mathematical model based on a proper decoupling approximation in order to investigate timely delivery ratio of the slotted Aloha with energy harvesting, not RF energy harvesting. In [29], we considered a cognitive radio network with RF energy harvesting where a primary node and a secondary node share a common channel, and the secondary node can harvest energy from the RF transmission signals of the primary node. We investigated an optimal sensing strategy exploiting channel usage pattern of the primary node.

The remainder of this paper is organized as follows. We present the system model in Section 2. In Section 3, we present two queueing models for our system. In Section 4, we develop mathematical models for analyzing the system. In Section 5, we derive the system throughput and the mean packet delay and then find the optimal transmission probabilities for optimizing the system throughput and the mean packet delay. Finally, Section 6 concludes this paper.

## 2. System Model

### 2.1. Network Model

**Figure 1:**
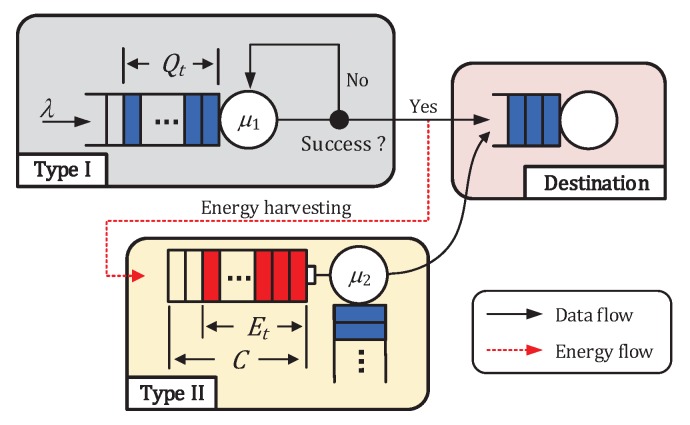
Original system model.

We consider a wireless network comprising two types of nodes, a Type I node and a Type II node, as shown in Figure 1. A slotted-Aloha multiple access channel is considered. A single wireless channel is time-slotted, and the two nodes are time-synchronized as assumed in [10,11,12,14,29] so that the slot duration is equal to the transmission time of one data packet. For each type of node, the following assumptions are made.

- The Type I node has a data queue for storing data packets and an unlimited energy supply from external energy sources. The capacity of the data queue is assumed to be infinite. Data packets arrive in the data queue according to a Bernoulli process with mean λ, 0<λ<1. That is, one data packet arrives with probability λ or not with probability 1−λ.
- The Type II node has an energy queue for storing energy packets and a backlogged (or saturated) data source. The capacity of the energy queue is assumed to be infinite (The assumption of infinite energy queue size is made for mathematical tractability. Our result serves as a bound for the case of finite energy queue.). We assume that for each data transmission the Type II node consumes m>1 energy packets, which is called *energy cost of transmission*.

The Type I node transmits a data packet in a slot with a probability $0<\mu_{1}\leq 1$ whenever its data queue is nonempty. The Type II node transmits a data packet in a slot with a probability $0<\mu_{2}\leq 1$ only when its energy queue has energy packets sufficient (not less than *m*) to send a data packet. We consider a simple collision channel model (Under the assumption of a channel model with multi-packet reception capability, e.g., a packet erasure channel model as in [11,12], simultaneous transmissions of multiple nodes may be successfully decoded by the receiver with a certain possibility. Extension to this more general channel model is a subject of future research issue.) as in [13,28,29], where the destination successfully decodes a transmitted data packet if only one node transmits. If two nodes transmit simultaneously, a collision occurs and both packets are lost. The collided packets must then be retransmitted in future time slots. For each successful transmission, the destination sends an immediate acknowledgment via an error-free feedback channel at the end of the slot.

**Remark** **1.** *A two-user slotted Aloha system with RF energy harvesting is also considered in [12]. Even for this small-sized network, the optimal aloha transmission probabilities of nodes while satisfying a given QoS constraint and the energy causality constraint has not been investigated so far. To extend the analysis to a bigger multi-user system presents serious difficulties of tractability due to the complex interactions between nodes and may require further approximations (e.g., [13,14]) or alternative approaches which go beyond the scope of this paper.*

### 2.2. RF Energy-Harvesting Model

We assume that the Type II node has no energy supply from external energy sources other than RF energy harvesting. The wireless channel shared between the Type I and II nodes is used not only for the data transmission of the Type II node, but also for energy harvesting from the RF signals transmitted by the Type I node, as assumed in [12,29]. The Type II node operates under a half-duplex mode, i.e., it can either harvest energy or transmit a data packet in each slot, but not both simultaneously. Under the half-duplex assumption, the energy-harvesting opportunities occur in the slots where the Type II node does not transmit while the Type I node is transmitting. Therefore, the Type II node cannot harvest energy when it attempts to transmit a data packet. We assume that the Type II node harvests a single energy packet with a fixed size from an RF transmission of the Type I node, if possible, as shown in Figure 1. It is assumed that *m* energy packets are consumed for a data packet transmission of the Type II node. It is worth elaborating our energy harvesting and consumption model.

- **Energy packet of fixed size**: The amount of energy in a single energy packet is defined as the average amount of energy harvested from an RF transmission of the Type I node. In general, the power received by the Type II node from the transmissions of the Type I node varies in each slot depending on several factors, such as the distance and RF harvesting efficiency [4]. Hence, the size of the energy packet depends on the efficiency of the harvesting antenna.
- **Energy cost of transmission *m***: For the transmission of a single data packet, the Type II node has to accumulate *m* energy packets. Thus, our energy consumption model is more general compared with those in [11,12] because the number of time slots required to harvest *m* energy packets is not geometrically distributed. Under the assumption of the fixed transmission power of the Type I node, i.e., no power control, a higher energy-harvesting efficiency leads to a larger energy packet size, thereby resulting in a smaller energy cost of transmission *m* needed for a single data packet transmission.

## 3. Queueing Model

**Figure 2:**
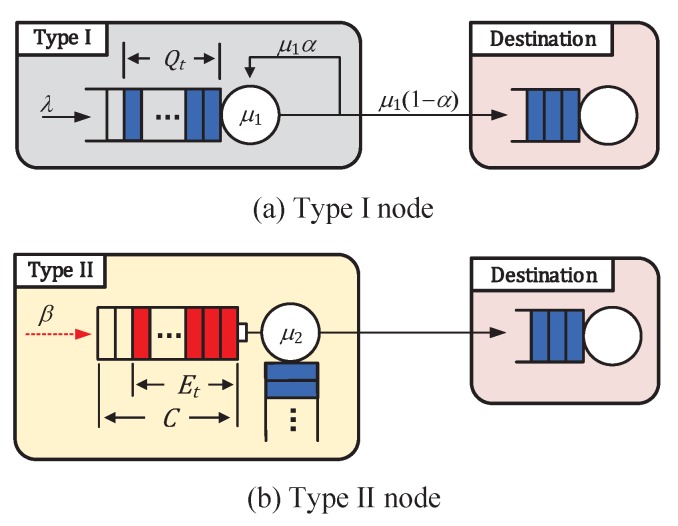
Modified system model.

We present two queueing models for the analysis: the original model (see Figure 1) and the modified model (see Figure 2). For analysis, the following assumptions are made.

- Data packet arrivals at the Type I node and energy packet arrivals at the Type II node occur at the beginning of slot *t*, if any.
- Data packet departures (service completion) from the Type I node and energy packet departures from the Type II node occur at the end of slot *t*, if any.
- We observe the system immediately before the end of slot *t*.

### 3.1. Exact Queueing Model

Let $Q_{t}$ and $E_{t}$ be the number of data packets in the data queue of the Type I node and the number of energy packets in the energy queue of the Type II node observed immediately before the end of slot *t*, respectively. Then, $Q_{t}\in \left\{0,1,2,\cdots \right\}$ and $E_{t}\in \left\{0,1,2,\cdots \right\}$. The evolution of $Q_{t}$ is governed by

$$Q_{t+1}=\max\left(0,Q_{t}-Y_{t}^{1}\right)+X_{t}^{1},$$

where $X_{t}^{1}\in \left\{0,1\right\}$ is the data packet arrival process of the Type I node and $Y_{t}^{1}\in \left\{0,1\right\}$ is the data packet departure process of the Type I node, which is defined as follows. $Y_{t}^{1}=1$ if all the following statements are true: (1) $Q_{t}>0$, (2) the Type I node attempts to transmit, and (3) either the Type II node does not attempt to transmit or $E_{t}<m$; otherwise, $Y_{t}=0$. Hence, it should be noted that the departure process $Y_{t}^{1}$ for the data queue depends on the energy queue status and the action (idle or transmitting) of the Type II node. The evolution of the energy queueing process $E_{t}$ is given by

$$E_{t+1}=\max\left(0,E_{t}-Y_{t}^{2}\right)+Y_{t}^{1},$$

where $Y_{t}^{2}\in \left\{0,m\right\}$ is the energy packet departure process of the Type II node, which is defined as follows: $Y_{t}^{2}=m$ (with the probability $\mu_{2}$) if the Type II node transmits a data packet; otherwise, $Y_{t}^{2}=0$ (with the probability $1-\mu_{2}$). We note that a data packet departure from the Type I node triggers an energy packet arrival to the Type II node.

The two processes $Q_{t}$ and $E_{t}$ are strongly coupled. The joint process $\left\{\left(Q_{t},E_{t}\right):\;t=0,1,2,\cdots \right\}$ is a discrete-time Markov chain. It is easy to see that this process is a quasi-birth-and-death process. Let $x_{i,j}=\lim_{t\to \infty }P\left(Q_{t}=i,E_{t}=j\right)$ (i≥0,j≥0) be the steady-state probability distribution of the Markov chain. The steady-state probability distribution can be readily obtained by applying the conventional matrix-analytic method, from which other performance measures can also be derived. This approach is computationally efficient, but it is difficult or impossible to obtain any insight into how the various system parameters affect the system performance. Instead of this approach, we resort to an approximate modeling approach.

### 3.2. An Approximate Model

The two stochastic processes $Q_{t}$ and $E_{t}$ in the original system interact with each other because the departure process from the data queue of the Type I node greatly depends on both the energy queue status $E_{t}$ and the action (transmitting or idle) of the Type II node. Furthermore, the energy arrival process for the energy queue of the Type II node is affected by both the data queue status $Q_{t}$ and the action of the Type I node. To relieve this strong dependency, we propose *a modified system* corresponding to the original system, which is shown in Figure 2 and will be explained below. In the modified system, the two queueing processes $Q_{t}$ and $E_{t}$ are loosely coupled via the parameters relating two processes, which will be presented below.

We define ${\hat{Q}}_{t}$ and ${\hat{E}}_{t}$ as the data queueing process for the Type I node and the energy queueing process of the Type II node in the modified system, respectively. The modified system based on a decoupling approximation is analyzed as follows.

- We first analyze the data queueing process ${\hat{Q}}_{t}$ of the Type I node under the assumption that the energy queueing process ${\hat{E}}_{t}$ of the Type II node is in a steady state (see Figure 2a). In order to consider the interaction between the data and energy queueing processes, we introduce the following probability:
  (1) $$\begin{array}{c} \alpha =1-P({\hat{E}}_{t}\geq m,\;\mathrm{Type}\;\mathrm{II}\;\mathrm{node}\;\mathrm{transmits}). \end{array}$$
  In what follows, we denote $\bar{x}=1-x$ for 0<x≤1. The probability $\bar{\alpha }=1-\alpha$ is the steady-state probability that the Type II node makes a transmission attempt in a slot, which can be written as follows:
  (2) $$\begin{array}{lll} \alpha & = & 1-P({\hat{E}}_{t}\geq m,\;\mathrm{Type}\;\mathrm{II}\;\mathrm{node}\;\mathrm{transmits})\\ & = & 1-P\left(\mathrm{Type}\;\mathrm{II}\;\mathrm{node}\;\mathrm{transmits}|{\hat{E}}_{t}\geq m\right)P\left({\hat{E}}_{t}\geq m\right)\\ & = & 1-\mu_{2}P\left({\hat{E}}_{t}\geq m\right). \end{array}$$
  In the original model, the data packet departure process of the Type I node is influenced by both the energy queue status and the action of the Type II node. However, in the modified system, the two stochastic processes ${\hat{Q}}_{t}$ and ${\hat{E}}_{t}$ are loosely coupled via the parameter α.
- Second, we separately analyze the energy queueing process ${\hat{E}}_{t}$ of the Type II node under the assumption that the data queueing process ${\hat{Q}}_{t}$ of the Type I node is in steady state (see Figure 2b). In order to consider the interaction between ${\hat{Q}}_{t}$ and ${\hat{E}}_{t}$, we introduce the following probability:
  (3) $$\begin{array}{c} \beta =P({\hat{Q}}_{t}\geq 1,\;\mathrm{Type}\;I\;\mathrm{node}\;\mathrm{transmits}), \end{array}$$
  which represents the steady-state probability that the Type I node has at least one data packet to send and it makes a transmission attempt in slot *t*. Then, β can be written as
  (4) $$\begin{array}{lll} \beta & = & P({\hat{Q}}_{t}\geq 1,\;\mathrm{Type}\;I\;\mathrm{node}\;\mathrm{transmits})\\ & = & \mu_{1}P\left({\hat{Q}}_{t}\geq 1\right) \end{array}$$

We denote the stationary distributions of ${\hat{Q}}_{t}$ and ${\hat{E}}_{t}$ by $y_{i}=\lim_{t\to \infty }P\left({\hat{Q}}_{t}=i\right)$, i≥0, and $z_{j}=\lim_{t\to \infty }P\left({\hat{E}}_{t}=j\right)$, j≥0, respectively.

In the remainder of this paper, the analysis is performed along the following steps:

1. For the modified system, we derive the stationary distributions $y_{i}$ and $z_{j}$.
2. The joint stationary probability $x_{i,j}$ for the original system $\left(Q_{t},E_{t}\right)$ is approximated by
  (5) $$\begin{array}{c} x_{i,j}\approx y_{i}\cdot z_{j}. \end{array}$$
3. Performance measures such as system throughput and mean packet delay are derived using the stationary distribution. Finally, we obtain the optimal transmission probabilities $\mu_{1}$ and $\mu_{2}$ which optimize the system throughput and the packet delay.

## 4. Analysis of the Modified System

In this section, we analyze the modified system. First, we derive the stationary distribution of the Markov chain $\left\{{\hat{Q}}_{t}:\;t=0,1,2,\cdots \right\}$ and then derive the stationary distribution of the Markov chain $\left\{{\hat{E}}_{t}:\;t=0,1,2,\cdots \right\}$.

### 4.1. Stationary Distribution of ${\hat{Q}}_{t}$

**Figure 3:**
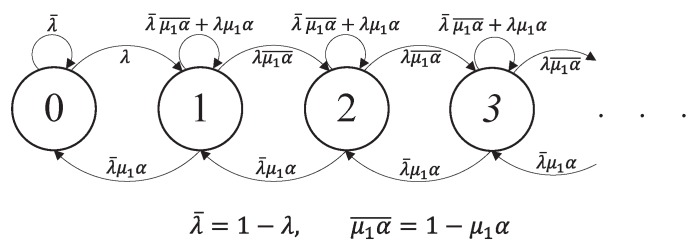
Markov chain model ${\hat{Q}}_{t}$ of the Type I node’s data queue, given that the Type II node’s energy queue ${\hat{E}}_{t}$ is in a steady state. Note that $\alpha =1-\mu_{2}P\left({\hat{E}}_{t}\geq m\right)$.

Suppose that the energy queueing process ${\hat{E}}_{t}$ of the Type II node is in a steady state. The one-step transition probabilities of ${\hat{Q}}_{t}$ are given as follows (see Figure 3).

- Given a current state ${\hat{Q}}_{t}=i>1$, ${\hat{Q}}_{t+1}=i-1$ when no data packet arrives with probability 1−λ and the Type I node transmits a data packet successfully with probability $\bar{\lambda }\mu_{1}\alpha$; ${\hat{Q}}_{t+1}=i+1$, when one data packet arrives with probability λ and no successful transmission occurs with probability $\lambda \bar{\mu_{1}\alpha }$, where $\bar{\mu_{1}\alpha }=1-\mu_{1}\alpha$; otherwise, ${\hat{Q}}_{t+1}=i$, with probability $\bar{\lambda }\bar{\mu_{1}\alpha }+\lambda \mu_{1}\alpha$.
- Given a current state ${\hat{Q}}_{t}=0$, ${\hat{Q}}_{t+1}=1$ when a data packet arrives with probability λ; otherwise, ${\hat{Q}}_{t+1}=0$.

The balance equations are given by

(6) $$\begin{array}{ccc} y_{0} & = & \lambda y_{0}+\left(\bar{\lambda }\mu_{1}\alpha \right)y_{1}\\ y_{i} & = & \left(\bar{\lambda }\bar{\mu_{1}\alpha }+\lambda \mu_{1}\alpha \right)y_{i}+\left(\lambda \bar{\mu_{1}\alpha }\right)y_{i-1} \end{array}$$

(7) $$\begin{array}{c} +\left(\bar{\lambda }\mu_{1}\alpha \right)y_{i+1},\;i\geq 1. \end{array}$$

Solving the balance Equations (6) and (7) yields

(8) $$\begin{array}{c} y_{i}={\left(\frac{\lambda \bar{\mu_{1}\alpha }}{\bar{\lambda }\mu_{1}\alpha }\right)}^{i}\frac{1}{\bar{\mu_{1}\alpha }}y_{0},\;i\geq 1. \end{array}$$

If $\lambda \bar{\mu_{1}\alpha }<\bar{\lambda }\mu_{1}\alpha$ $\left(\Leftrightarrow \lambda <\mu_{1}\alpha \right)$, then the normalization condition $\sum_{i=0}^{\infty }y_{i}=1$ leads to $y_{0}=1-\frac{\lambda }{\mu_{1}\alpha }.$ Hence, the stationary distribution of the Markov chain is given by

(9) $$\begin{array}{ccc} y_{i} & = & {\left(\frac{\lambda \bar{\mu_{1}\alpha }}{\bar{\lambda }\mu_{1}\alpha }\right)}^{i}\frac{1}{\bar{\mu_{1}\alpha }}y_{0},\;i\geq 1. \end{array}$$

(10) $$\begin{array}{ccc} y_{0} & = & 1-\frac{\lambda }{\mu_{1}\alpha }. \end{array}$$

### 4.2. Stationary Distribution of ${\hat{E}}_{t}$

**Figure 4:**
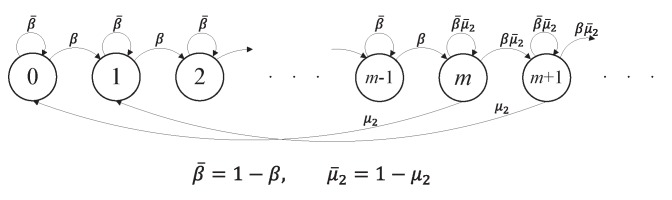
Markov chain model ${\hat{E}}_{t}$ of the Type II node’s energy queue, given that the Type I node’s data queue ${\hat{Q}}_{t}$ is in a steady state. Note that $\beta =\mu_{1}P\left({\hat{Q}}_{t}\geq 1\right)$.

Suppose that the data queueing process ${\hat{Q}}_{t}$ of the Type I node is in a steady state. The one-step transition probabilities of the Markov chain $\left\{{\hat{E}}_{t}:\;t=0,1,2,\cdots \right\}$ are given as follows (see Figure 4).

- Given a current state ${\hat{E}}_{t}=j$ for j≥m, ${\hat{E}}_{t}=j+1$ (with probability β) when the Type II node does not make a transmission attempt and the Type I node makes a transmission; ${\hat{E}}_{t}=j-m$ (with probability $\mu_{2}$) when the Type II node makes a transmission; and ${\hat{E}}_{t}=j$ (with probability $\bar{\beta }{\bar{\mu }}_{2}$) when both the Type I and Type II nodes do not attempt to transmit.
- Given a current state ${\hat{E}}_{t}=j$ for 0≤j≤m−1, ${\hat{E}}_{t}=j+1$ (with probability β) when the Type I node makes a transmission; and ${\hat{E}}_{t}=j$ (with probability $\bar{\beta }$) when the Type I node does not attempt to transmit.

Thus, the balance equations for the Markov chain are given as follows:

(11) $$\begin{array}{ccc} z_{0}\;\;\; & = & \;\;\;\bar{\beta }z_{0}+\mu_{2}z_{m} \end{array}$$

(12) $$\begin{array}{ccc} z_{j}\;\;\; & = & \;\;\;\bar{\beta }z_{j}+\beta z_{j-1}+\mu_{2}z_{j+m},\;\mathrm{for}\;1\leq j\leq m-1 \end{array}$$

(13) $$\begin{array}{ccc} z_{m}\;\;\; & = & \;\;\;\bar{\beta }\bar{\mu_{2}}z_{m}+\beta z_{m-1}+\mu_{2}z_{2m} \end{array}$$

(14) $$\begin{array}{ccc} z_{j}\;\;\; & = & \;\;\;\bar{\beta }\bar{\mu_{2}}z_{j}+\beta \bar{\mu_{2}}z_{j-1}+\mu_{2}z_{j+m},\;\mathrm{for}\;j\geq m+1. \end{array}$$

We first guess a geometric-form solution for the recurrence relation (14), as follows:

(15) $$\begin{array}{c} z_{j}=b\left(1-z\right)z^{j-m},\;j\geq m, \end{array}$$

where *b* is a constant that will be determined later and *z* is a solution of the following equation

(16) $$\begin{array}{c} \mu_{2}z^{m+1}-\left(1-\bar{\beta }\bar{\mu_{2}}\right)z+\beta \bar{\mu_{2}}=0. \end{array}$$

Note that (16) satisfies (14). Let $f\left(z\right)=\mu_{2}z^{m+1}-\left(1-\bar{\beta }\bar{\mu_{2}}\right)z+\beta \bar{\mu_{2}}$. Note that f(0)>0, f(1)=0, $f^{\prime \prime }\left(z\right)>0$. Thus, if $f^{\prime }\left(1\right)=\left(m+\beta \right)\mu_{2}-\beta >0$, then (16) has a unique solution in the interval (0,1).

Using (15), we can rewrite the balance Equations (11)–(14) as

(17) $$\begin{array}{ccc} z_{0} & = & \frac{\mu_{2}}{\beta }b\left(1-z\right) \end{array}$$

(18) $$\begin{array}{ccc} z_{j} & = & \frac{\mu_{2}}{\beta }b\left(1-z^{j+1}\right),\;\mathrm{for}\;1\leq j\leq m-1 \end{array}$$

(19) $$\begin{array}{ccc} z_{j} & = & b\left(1-z\right)z^{j-m},\;\mathrm{for}\;j\geq m. \end{array}$$

Summing up $z_{j}$ from j=0 to j=m−1, we have

(20) $$\begin{array}{lll} \sum_{j=0}^{m-1}z_{j} & = & \frac{\mu_{2}}{\beta }b\sum_{j=0}^{m-1}\left(1-z^{j+1}\right)\\ & = & \frac{\mu_{2}}{\beta }b\left(m-\sum_{j=0}^{m-1}z^{j+1}\right)\\ & = & \frac{\mu_{2}}{\beta }b\left(m-\frac{\beta \bar{\mu_{2}}}{\mu_{2}}\right), \end{array}$$

where the last equality comes from the fact that factoring (16) yields $\left(z-1\right)\left(\mu_{2}\sum_{j=0}^{m-1}z^{j+1}-\beta \bar{\mu_{2}}\right)=0$ and 0<z<1 is a solution of equation $\mu_{2}\sum_{j=0}^{m-1}z^{j+1}-\beta \bar{\mu_{2}}=0$. Next, we have

(21) $$\begin{array}{c} \sum_{j=m}^{\infty }z_{j}=\sum_{j=m}^{\infty }b\left(1-z\right)z^{j-m}=b. \end{array}$$

From (20) and (21), the normalization condition $\sum_{j}z_{i}=1$ yields

(22) $$\begin{array}{c} b=\frac{1}{\mu_{2}\left(m/\beta +1\right)}. \end{array}$$

We now summarize the results in the following theorem.

**Theorem** **1.** *If $\left(m+\beta \right)\mu_{2}>\beta$, then the Markov chain $\left\{{\hat{E}}_{t}:\;t=0,1,2,\cdots \right\}$ is positive recurrent and the steady-state distribution $z_{j}\;\left(j\geq 0\right)$ is obtained as follows:*

(23) $$\begin{array}{ccc} z_{j} & = & \frac{1}{m+\beta }\left(1-z^{j+1}\right),\;for\;0\leq j\leq m-1 \end{array}$$

(24) $$\begin{array}{ccc} z_{j} & = & \frac{1}{\mu_{2}\left(m/\beta +1\right)}\left(1-z\right)z^{j-m},\;for\;j\geq m, \end{array}$$

*where 0<z<1 is a solution of the Equation (16).*

## 5. Optimal Transmission Probabilities

In this section, we find the optimal transmission probabilities $\mu_{1}$ and $\mu_{2}$ which minimize the mean sojourn time of a data packet for the Type II node while maximizing system throughput. For this purpose, we first derive the key probabilities α and β using the steady-state distribution of the Markov chain derived in the previous Section. Specifically, this is carried out for the two cases: Case (1) the Type I node is in the energy-limited phase and Case (2) the Type II node is in the energy-unlimited phase. For each case, we derive the maximum system throughput and the corresponding optimal transmission probabilities. We derive the mean sojourn time of a data packet for the Type I node. Finally, we find the optimal transmission probabilities to minimize the mean sojourn time while maximizing the system throughput.

### 5.1. Derivation of α and β

In the rest of this paper, we always impose the data queue of the Type I node to satisfy the stability condition $\lambda <\mu_{1}\alpha$. The energy queue of the Type II node can be in two phases by controlling the transmission probability $\mu_{2}$. A large transmission probability $\mu_{2}$ can be used to consume most harvested energy and thus keep the energy queue small. On the other hand, with a small transmission probability $\mu_{2}$, the Type II node consumes less energy and thus the energy queue may be in unstable phase, i.e., grow explosively. It is not clear what would be better from a perspective of system throughput or delay. For this reason, under the condition that the data queue of the Type I node is stable, we consider two cases depending on the phase that the Type II node can select.

- **Case (1) Energy-limited phase**: This case represents the situation that the energy queue is stable, i.e., satisfies the condition $\left(m+\beta \right)\mu_{2}>\beta$ in the Theorem 1. In this case, the Type II node consumes its energy actively for its data transmission. As a result, the Type II node lacks energy.
- **Case (2) Energy-unlimited phase**: This case indicates the situation that the energy queue is unstable, i.e., $\left(m+\beta \right)\mu_{2}\leq \beta$. The Type II node consumes less energy and the energy queue grows explosively.

We derive the probabilities α and β for two cases, respectively.

#### 5.1.1. Case (1) Energy-Limited Phase

Suppose that the energy queue is stable, $\left(m+\beta \right)\mu_{2}>\beta$. The probabilities α and β are expressed in terms of the stationary distributions $y_{i}$ and $z_{j}$. Noting that from (24) $P\left({\hat{E}}_{t}\geq m\right)=\sum_{j=m}^{\infty }z_{j}=\frac{1}{\mu_{2}\left(m/\beta +1\right)}$, α is derived as

(25) $$\begin{array}{ccc} \alpha & = & 1-\mu_{2}P\left({\hat{E}}_{t}\geq m\right)=1-\frac{1}{m/\beta +1}. \end{array}$$

In addition, $P\left({\hat{Q}}_{t}\geq 1\right)=1-y_{0}=\frac{1}{\mu_{1}\alpha }$ from (9), and thus β is obtained as

(26) $$\begin{array}{ccc} \beta & = & \mu_{1}P\left({\hat{Q}}_{t}\geq 1\right)=\frac{\lambda }{\alpha }. \end{array}$$

Solving (25) and (26) leads to

(27) $$\begin{array}{ccc} \alpha & = & \frac{m-\lambda }{m} \end{array}$$

(28) $$\begin{array}{ccc} \beta & = & \frac{m\lambda }{m-\lambda }\;\mathrm{for}\;\lambda \leq \frac{m}{m+1}. \end{array}$$

We also have from (25) and (26) that the stability condition $\lambda <\mu_{1}\alpha$ for the data queue is equivalent to $\frac{m\lambda }{m-\lambda }<\mu_{1}\leq 1$ and the stability condition $\left(m+\beta \right)\mu_{2}>\beta$ for the energy queue is equivalent to $\frac{\lambda }{m}<\mu_{2}\leq 1$. Summarizing the results, we have the following.

**Lemma** **1.** *If both the energy queue and the date queue are stable, that is, $\frac{m\lambda }{m-\lambda }<\mu_{1}\leq 1$ and $\frac{\lambda }{m}<\mu_{2}\leq 1$, then we have*

$$\begin{array}{ccc} \alpha & = & \frac{m-\lambda }{m}\\ \beta & = & \frac{m\lambda }{m-\lambda }\;for\;\lambda \leq \frac{m}{m+1}. \end{array}$$

**Remark** **2.** *Lemma 1 indicates that the maximum arrival rate for data packets of the Type I node should not be greater than $\frac{m}{m+1}$ for stabilizing two queues simultaneously, which can be interpreted as follows: with a large arrival rate λ, the transmission probability $\mu_{1}$ should be larger to ensure the stability of the data queue. Then, the Type II node has more opportunities to harvest energy, which results in a large transmission probability $\mu_{2}$ for the stability of the energy queue and hence the maximum arrival rate is restricted by the collision effect.*

#### 5.1.2. Case (2) Energy-Unlimited Phase

Suppose that the energy queue is unstable, i.e., $\left(m+\beta \right)\mu_{2}\leq \beta$. Then, the energy queue grows to infinity and thus $P({\hat{E}}_{t}\geq m)=1$. α is therefore obtained as

(29) $$\begin{array}{ccc} \alpha & = & 1-\mu_{2}P\left({\hat{E}}_{t}\geq m\right)=1-\mu_{2}. \end{array}$$

Similar to (26), β is obtained as

(30) $$\begin{array}{ccc} \beta & = & \frac{\lambda }{\alpha }=\frac{\lambda }{1-\mu_{2}}. \end{array}$$

It is therefore seen that the stability condition $\lambda <\mu_{1}\alpha$ for the data queue is equivalent to $\lambda <\mu_{1}\left(1-\mu_{2}\right)$ and the condition $\left(m+\beta \right)\mu_{2}\leq \beta$ for the energy queue is equivalent to $\mu_{2}\leq \frac{\lambda }{m}$. Summarizing the results, we have the following.

**Lemma** **2.** *If the data queue is stable, i.e., $\lambda <\mu_{1}\left(1-\mu_{2}\right)$, and the energy queue is unstable, i.e., $\mu_{2}\leq \frac{\lambda }{m}$, we then have*

$$\begin{array}{ccc} \alpha & = & 1-\mu_{2}\\ \beta & = & \frac{\lambda }{1-\mu_{2}}. \end{array}$$

### 5.2. Maximum System Throughput

In this section, we derive the maximum system throughput and the corresponding optimal transmission probabilities for two cases, respectively. Suppose that the modified system is in a steady state in slot *t*. We define the system throughput, denoted by *T*, as the number of data packets transmitted in a slot. Thus, the system throughput is not greater than 1. We define $T_{i}$ as the throughput of the Type *i* node, i= I, II. Then, $T=T_{I}+T_{II}$.

#### 5.2.1. Case (1) Energy-Limited Phase

Suppose that both the data queue and the energy queue are stable, $\frac{m\lambda }{m-\lambda }<\mu_{1}\leq 1$ and $\frac{\lambda }{m}<\mu_{2}\leq 1$. $T_{I}$ is given by

(31) $$\begin{array}{ccc} T_{I} & = & \beta \alpha . \end{array}$$

$T_{II}$ is derived as

(32) $$\begin{array}{ccc} T_{II} & = & (1-\alpha )(1-\beta ). \end{array}$$

Using (27), (28), (31), and (32), the system throughput is given in the following Theorem.

**Theorem** **2.** *For any $\frac{m\lambda }{m-\lambda }<\mu_{1}\leq 1$ and $\frac{\lambda }{m}<\mu_{2}\leq 1$, $T_{I}$, $T_{II}$ and T are derived as*

(33) $$\begin{array}{c} T_{I}=\lambda ,\;T_{II}=\frac{\lambda }{m}\left(1-\frac{\lambda }{1-\lambda /m}\right) \end{array}$$

(34) $$\begin{array}{c} T=\lambda +\frac{\lambda }{m}\left(1-\frac{\lambda }{1-\lambda /m}\right). \end{array}$$

**Remark** **3.** *From Theorem 2, we can deduce the following.*

- *The system throughput is insensitive to the transmission probabilities $\mu_{1}$ and $\mu_{2}$ if $\mu_{1}>\frac{m\lambda }{m-\lambda }$ and $\mu_{2}>\frac{\lambda }{m}$. Thus, for a given arrival rate λ, the maximum system throughput is the same regardless of $\mu_{1}$ and $\mu_{2}$. This implies that choosing $\mu_{1}=1$ is most desirable from the perspective of packet delay of the Type I node, as will be discussed in the next section.*
- *$T_{I}=\lambda$ and $T_{II}=\frac{\lambda }{m}\left(1-\frac{\lambda m}{m-\lambda }\right)$ provided that both queues are stable. For a given λ, the throughput $T_{I}$ of the Type I node is always equal to λ as long as the data queue is stable. On the other hand, the throughput $T_{II}$ of the Type II node decreases with m.*

#### 5.2.2. Case (2) Energy-Unlimited Phase

Suppose that the data queue is stable and the energy queue is not stable, $\lambda <\mu_{1}\left(1-\mu_{2}\right)$ and $\mu_{2}\leq \frac{\lambda }{m}$. In a similar way to Case (1), we have $T_{I}=\beta \alpha$ and $T_{II}=\left(1-\alpha \right)\left(1-\beta \right)$. Combining this with (29) and (30) leads to the following.

**Theorem** **3.** *Suppose that $\lambda <\mu_{1}\left(1-\mu_{2}\right)$ and $\mu_{2}\leq \frac{\lambda }{m}$.*

1. *The system throughput is derived as*
(35) $$\begin{array}{c} T_{I}=\lambda ,\;T_{II}=\mu_{2}\left(1-\frac{\lambda }{1-\mu_{2}}\right) \end{array}$$
(36) $$\begin{array}{c} T=\lambda +\mu_{2}\left(1-\frac{\lambda }{1-\mu_{2}}\right). \end{array}$$
2. *The maximum system throughput $T^{*}$ and the corresponding optimal transmission probabilities $\mu_{1}^{*}$ and $\mu_{2}^{*}$ are obtained as follows:*
  - *If $\frac{\lambda }{m}\leq 1-\sqrt{\lambda }$, then*
(37) $$\begin{array}{c} T^{*}=\lambda +\frac{\lambda }{m}\left(1-\frac{\lambda m}{m-\lambda }\right) \end{array}$$
(38) $$\begin{array}{c} \mu_{1}^{*}>\frac{\lambda }{1-\lambda /m},\;\mu_{2}^{*}=\frac{\lambda }{m}. \end{array}$$
  - *If $\frac{\lambda }{m}>1-\sqrt{\lambda }$, then*
(39) $$\begin{array}{c} T^{*}=\lambda +{\left(1-\sqrt{\lambda }\right)}^{2} \end{array}$$
(40) $$\begin{array}{c} \mu_{1}^{*}>\sqrt{\lambda },\;\mu_{2}^{*}=1-\sqrt{\lambda }. \end{array}$$

**Proof.**  Let $f\left(\mu_{2}\right)=\mu_{2}\left(1-\frac{\lambda }{1-\mu_{2}}\right)$. Differentiating $f(\mu_{2})$ with respect to $\mu_{2}$, we have $\frac{df(\mu_{2})}{d\mu_{2}}=\frac{\mu_{2}^{2}-2\mu_{2}+1-\lambda }{{\left(1-\mu_{2}\right)}^{2}}=\frac{\left(\mu_{2}-\left(1-\sqrt{\lambda }\right)\right)\left(\mu_{2}-\left(1+\sqrt{\lambda }\right)\right)}{{\left(1-\mu_{2}\right)}^{2}}$. Noting that $\mu_{2}\leq \frac{\lambda }{m}\leq 1$, $\frac{df(\mu_{2})}{d\mu_{2}}=0$ leads to $\mu_{2}=1-\sqrt{\lambda }$. If $\frac{\lambda }{m}\leq 1-\sqrt{\lambda }$, $f(\mu_{2})$ is an increasing function of $\mu_{2}$ for $0<\mu_{2}\leq \frac{\lambda }{m}$ and thus has the global maximum at $\mu =\frac{\lambda }{m}$. Hence, (37) and (38) are derived. On the other hand, if $\frac{\lambda }{m}>1-\sqrt{\lambda }$, then $f(\mu_{2})$ increases for $\mu_{2}<1-\sqrt{\lambda }$ and then decreases for $1-\sqrt{\lambda }\leq \mu_{2}\leq \frac{\lambda }{m}$. Hence, $f(\mu_{2})$ has the global maximum at $\mu_{2}=1-\sqrt{\lambda }$ and (39) and (40) are derived. □

### 5.3. Discussions

In this section, based on Theorems 2 and 3, we discuss the achievable maximum throughput and the corresponding optimal transmission probabilities depending on the arrival probability λ. From Theorems 2 and 3, we have the following result.

**Corollary** **1.** *Let the energy cost of transmission m be fixed. The maximum system throughput $T^{*}$ and the corresponding optimal transmission probabilities $\mu_{1}^{*}$ and $\mu_{2}^{*}$ depending on the arrival rate 0<λ<1 are given as follows:*

- *If $\frac{\lambda }{m}\leq 1-\sqrt{\lambda }\;\left(\Leftrightarrow \lambda \leq \frac{2m}{m+2+\sqrt{m^{2}+4m}}\right)$, then $T^{*}=\lambda +\frac{\lambda }{m}\left(1-\frac{m\lambda }{m-\lambda }\right)$, $\mu_{1}^{*}>\frac{m\lambda }{m-\lambda }$ and $\mu_{2}^{*}\geq \frac{\lambda }{m}$.*
- *If $\frac{\lambda }{m}>1-\sqrt{\lambda }\;\left(\Leftrightarrow \lambda >\frac{2m}{m+2+\sqrt{m^{2}+4m}}\right)$, then $T^{*}=\lambda +{\left(1-\sqrt{\lambda }\right)}^{2}$, $\mu_{1}^{*}>\sqrt{\lambda }$, and $\mu_{2}^{*}=1-\sqrt{\lambda }$.*

**Proof.**  Let 0<λ<1 be given. It is easy to note that $\frac{\lambda }{m}\leq 1-\sqrt{\lambda }$ is equivalent to $\lambda \leq \frac{2m}{m+2+\sqrt{m^{2}+4m}}$. We also note that $\frac{2m}{m+2+\sqrt{m^{2}+4m}}<\frac{m}{m+1}$. Hence, we have the desired results from the Theorems 2 and 3. □

From this Corollary, we can deduce the following facts.

- **The maximum arrival rate**$\lambda \leq \frac{m}{m+1}$**for the stability of both the data queue and the energy queue in the Case (1)**: According to Theorem 2, the maximum arrival rate λ is bounded above $\frac{m}{m+1}$ for the stability of both the data queue and the energy queue. When the Type II node operates in the energy-limited phase, the throughput of Type II node approaches to zero as λ increases to $\frac{m}{m+1}$.
- **The energy-limited phase is better when**$\frac{\lambda }{m}\leq 1-\sqrt{\lambda }\Leftrightarrow \lambda \leq \frac{2m}{m+2+\sqrt{m^{2}+4m}}$: It is easy to see that 1) $\frac{\lambda }{m}\leq 1-\sqrt{\lambda }$ is equivalent to $\lambda \leq \frac{2m}{m+2+\sqrt{m^{2}+4m}}$ and 2) $\frac{2m}{m+2+\sqrt{m^{2}+4m}}<\frac{m}{m+1}$. Thus, when $\lambda \leq \frac{2m}{m+2+\sqrt{m^{2}+4m}}$, that is, the arrival rate λ is relatively low, it is beneficial for the Type I node to transmit data packets actively with a large $\mu_{1}$ and for the Type II node to attempt transmissions actively so that the energy queue does not grow.
- **The energy-unlimited phase is better when**$\frac{\lambda }{m}>1-\sqrt{\lambda }\Leftrightarrow \lambda >\frac{2m}{m+2+\sqrt{m^{2}+4m}}$: It is shown in Theorems 2 and 3 that when $\frac{2m}{m+2+\sqrt{m^{2}+4m}}<\lambda <\frac{m}{m+1}$, the Type II node may operate in either the energy-limited phase or the energy-unlimited phase. Corollary 1 tells us that it is better for the Type II node to be in the energy-unlimited phase in terms of maximizing the system throughput.

**Figure 5:**
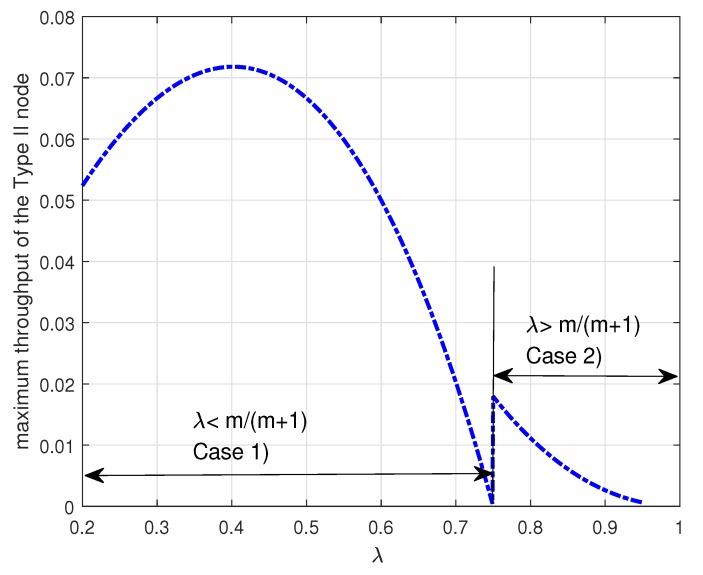
Maximum throughput $T_{II}^{*}$ of the Type II node for Case (1) and Case (2) when m=3. In this figure, Case (1) represents the maximum throughput of the Type II node given by the Theorem 2 and Case (2) represents the maximum throughput of the Type II node given by the Theorem 3.

**Figure 6:**
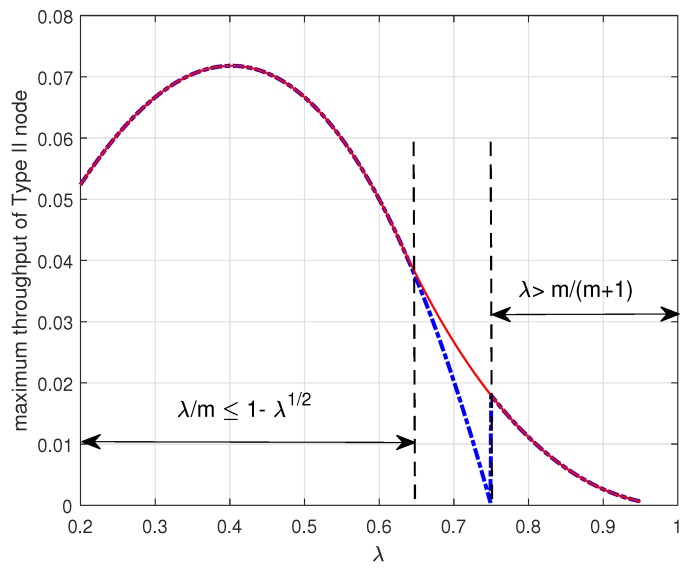
Maximum throughput $T_{II}^{*}$ of the Type II node attained by controlling the transmission probability $\mu_{2}$ depending on the packet arrival probability λ when m=3. The red solid line in the figure denotes the maximum throughput of the Type II node given by the Corollary 1.

Figure 5 shows the maximum throughput $T_{II}^{*}$ of the Type II node versus the arrival probability λ when m=3. In the figure, Case (1) denotes the maximum throughput $T_{II}^{*}$ given by the Theorem 2 and Case (2) denotes the maximum throughput $T_{II}^{*}$ given by the Theorem 3. In Figure 5, it is worth noting that the maximum throughput of the Type II node depends on the arrival rate λ as expected. In addition, we notice that the curve has a discontinuity at $\lambda =\frac{m}{m+1}$ and the maximum throughput of the Type II node decreases to zero as the arrival rate λ approaches to $\lambda =\frac{m}{m+1}$. As discussed above, if $\frac{\lambda }{m}\leq 1-\sqrt{\lambda }$ and $\lambda \leq \frac{m}{m+1}$, i.e., $\frac{2m}{m+2+\sqrt{m^{2}+4m}}<\lambda <\frac{m}{m+1}$, the Type II node may be in either the energy-limited phase (Case (1)) or the energy-unlimited phase (Case (2)). Figure 6 shows that it is better for the Type II node to be in the energy-unlimited phase so as to maximize its throughput. This result indicates that when the Type II node is allowed to be in two phases it is better for the Type II node to limit energy usage even if it has enough energy in the energy queue. Finally, we observe that there is an optimal arrival probability λ which maximizes the throughput of the Type II node.

**Figure 7:**
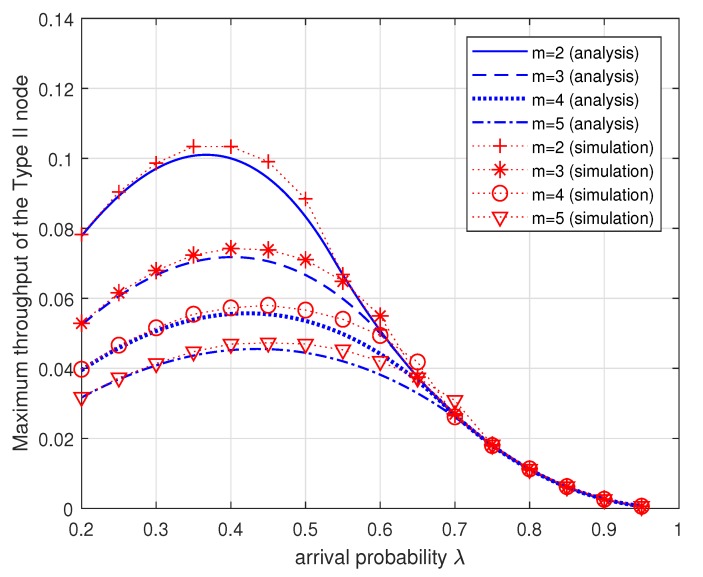
Maximum throughput $T_{II}^{*}$ of the Type II node (given by the Corollary 1) versus the arrival probability λ for different energy cost of transmission *m*.

In Figure 7, the maximum throughput $T_{II}^{*}$ of the Type II node is plotted for the varying arrival probability λ when m=2,3,4,5. For each λ, the maximum throughput $T_{II}^{*}$ of the Type II node is given by the Corollary 1. Note that the throughput of Type I node is always equal to the arrival probability λ as long as the data queue is stable. Simulation results are also plotted to validate the accuracy of our proposed analytical model. Simulations are performed using the Matlab software. Each simulation result shown in the figures is a result of performing for one million slots including an initial warm-up time of 0.3 million slots. Figure 7 shows that for each *m* the maximum throughput of the Type II node is dependent on the arrival probability λ and there exists an optimal λ which maximizes the throughput of the Type II node. We also see that as *m* increases the maximum throughput of the Type II node decreases as expected because more energy is necessary for a data packet transmission. We notice that when the arrival probability λ is relatively large, that is, the Type II node is in the energy-unlimited phase, the maximum throughput is almost same regardless of *m*. The analytical results and the simulation results agree within a reasonable accuracy.

**Figure 8:**
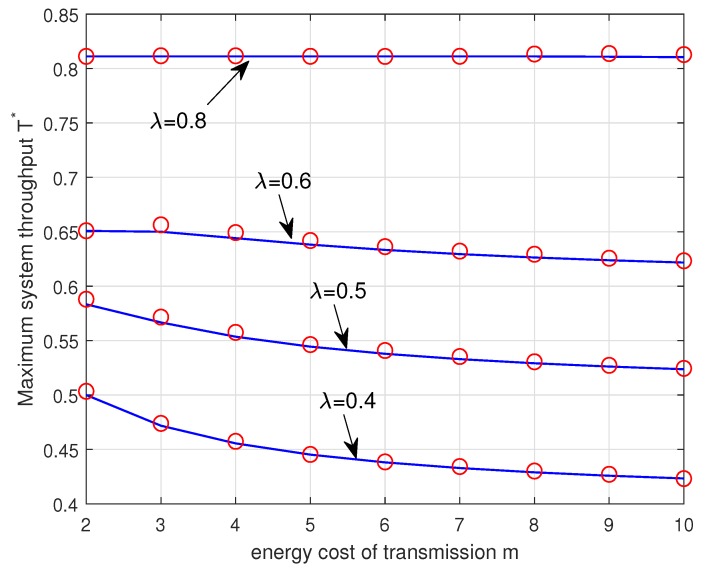
Maximum system throughput $T^{*}$ (given by the Corollary 1) versus the energy cost of transmission *m* for different λ=0.4,0.5,0.6,0.8. The analytical results and the simulation results are plotted in solid lines and ‘∘’ markers, respectively.

Figure 8 plots the maximum system throughput $T^{*}$ versus the energy cost of transmission *m* for different values of λ. The maximum system throughput $T^{*}$ and the corresponding optimal transmission probabilities $\mu_{1}^{*}$ and $\mu_{2}^{*}$ are given by the Corollary 1. It is seen that the maximum system throughput decreases with the increase of *m* and the decreasing rate in the maximum system throughput diminishes as the arrival probability λ increases. When λ=0.8, the maximum system throughput is the same regardless of *m*. In this case, the Type II node should be in the energy-unlimited phase so as to stabilize the data queue of the Type II node and therefore the throughput of the Type I node is constant even if it has enough energy in its energy queue. We again note that the analytical results match well with the simulation results.

### 5.4. Joint Optimization of Mean Sojourn Time and System Throughput

We derive the mean queue size, denoted by *L*, which is the average number of data packets in the Type I node’s data queue, including the packet in service. From (9), we have

$$\begin{array}{lll} L & = & \sum_{i=1}^{\infty }iy_{i}\\ & = & \sum_{i=1}^{\infty }i{\left(\frac{\lambda \bar{\mu_{1}\alpha }}{\bar{\lambda }\mu_{1}\alpha }\right)}^{i}\frac{1}{\bar{\mu_{1}\alpha }}y_{0}\\ & = & \frac{\lambda (1-\lambda )}{\mu_{1}\alpha -\lambda }. \end{array}$$

Let *W* be the mean sojourn time of a data packet, which is the sum of the waiting time in the queue before service and its service time. From Little’s formula L=λW, *W* is given by

(41) $$\begin{array}{c} W=\frac{1-\lambda }{\mu_{1}\alpha -\lambda }. \end{array}$$

Plugging (27) and (29), respectively, into (41), for two cases the mean sojourn time is derived as follows:

- **Mean sojourn time for Case (1)**: In this case, $\alpha =\frac{m-\lambda }{m}$ and thus
  (42) $$\begin{array}{c} W=\frac{1-\lambda }{\mu_{1}\left(\frac{m-\lambda }{m}\right)-\lambda }. \end{array}$$
  From the Theorem 2, the system throughput is maximized with any value $\frac{m\lambda }{m-\lambda }<\mu_{1}\leq 1$ and $\frac{\lambda }{m}<\mu_{2}\leq 1$. It is interesting to note that the mean sojourn time is insensitive to the transmission probability $\mu_{2}$ for $\mu_{2}>\frac{\lambda }{m}$ and is a decreasing function of $\mu_{1}$ for $\mu_{1}>\frac{m\lambda }{m-\lambda }$. Hence, it is desirable to choose $\mu_{1}=1$ to minimize the mean sojourn time while maximizing the system throughput.
- **Mean sojourn time for Case (2)**: In this case, $\alpha =1-\mu_{2}$ and hence
  (43) $$\begin{array}{c} W=\frac{1-\lambda }{\mu_{1}\left(1-\mu_{2}\right)-\lambda }. \end{array}$$
  It is worth noting that the mean sojourn time decreases as $\mu_{1}$ increases and $\mu_{2}$ decreases. In order to minimize the mean sojourn time while maximizing system throughput, it is desirable from the Theorem 3 to choose $\mu_{1}=1$ and $\mu_{2}=\frac{\lambda }{m}$ when $\frac{\lambda }{m}\leq 1-\sqrt{\lambda }$, and $\mu_{1}=1$ and $\mu_{2}=1-\sqrt{\lambda }$ when $\frac{\lambda }{m}>1-\sqrt{\lambda }$.
  Summarizing the Corollary 1, (42) and (42), we have the following result.

**Corollary** **2.** *Let the energy cost of transmission m be fixed. The optimal transmission probabilities $\mu_{1}^{*}$ and $\mu_{2}^{*}$ to minimize the mean sojourn time while maximizing the system throughput are given as follows:*

- *If $\frac{\lambda }{m}\leq 1-\sqrt{\lambda }$, i.e., $\lambda \leq \frac{2m}{m+2+\sqrt{m^{2}+4m}}$, then the minimum mean sojourn time $W^{*}$ is $W^{*}=\frac{1-\lambda }{\frac{m-\lambda }{m}-\lambda }$ for the optimal transmission probabilities $\mu_{1}^{*}=1$ and $\mu_{2}>\frac{\lambda }{m}.$*
- *If $\frac{\lambda }{m}>1-\sqrt{\lambda }$, i.e., $\lambda >\frac{2m}{m+2+\sqrt{m^{2}+4m}}$, then $W^{*}=\frac{1+\sqrt{\lambda }}{\sqrt{\lambda }}$ for the optimal transmission probabilities $\mu_{1}^{*}=1$ and $\mu_{2}^{*}=1-\sqrt{\lambda }$.*

**Figure 9:**
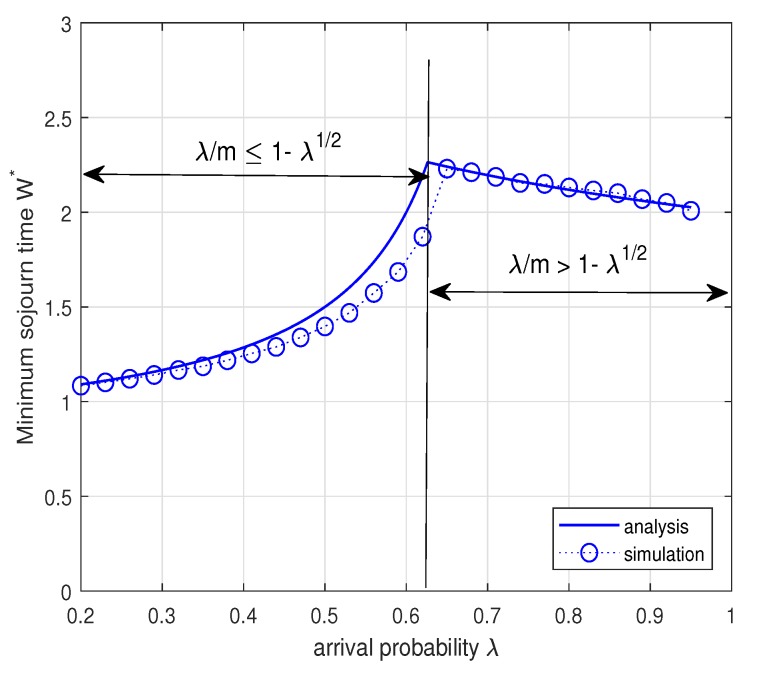
Minimum mean sojourn time $W^{*}$ while maximizing the system throughput for the varying λ when m=3. According to the Corollary 2, we set $\mu_{1}=1$ and $\mu_{2}=1$ when $\frac{\lambda }{m}\leq 1-\sqrt{\lambda }$ and $\mu_{1}=1$ and $\mu_{2}=1-\sqrt{\lambda }$ when $\frac{\lambda }{m}>1-\sqrt{\lambda }$.

**Figure 10:**
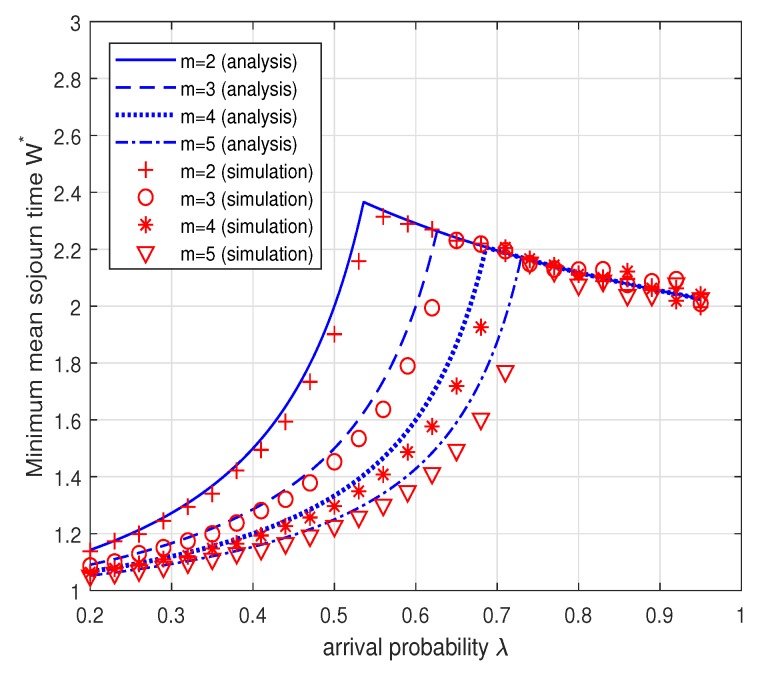
Minimum mean sojourn time $W^{*}$ while maximizing the system throughput versus λ for different m=2,3,4,5. According to the Corollary 2, we set $\mu_{1}=1$ and $\mu_{2}=1$ when $\frac{\lambda }{m}\leq 1-\sqrt{\lambda }$ and $\mu_{1}=1$ and $\mu_{2}=1-\sqrt{\lambda }$ when $\frac{\lambda }{m}>1-\sqrt{\lambda }$.

In the above Corollary, we note that the minimum mean sojourn time increases as λ increases to $\frac{2m}{m+2+\sqrt{m^{2}+4m}}$ and then decreases as λ increases from $\frac{2m}{m+2+\sqrt{m^{2}+4m}}$ to 1. The decrease in the mean sojourn time when the arrival rate increases seems to be against our intuition. This result can be interpreted as follows: (1) when $\frac{\lambda }{m}\leq 1-\sqrt{\lambda }$, the Type II node operates in the energy-limited phase with a relatively large transmission probability $\mu_{2}$ for stabilizing its energy queue and thus the sojourn time of a data packet for the Type I node is likely to increase due to collisions with the Type II node; (2) when $\frac{\lambda }{m}>1-\sqrt{\lambda }$, the Type II node should maintain a small transmission probability $\mu_{2}$ for stabilizing the data queue of the Type II node and thus the possibility of collision decreases, which results in smaller mean sojourn time. This interpretation is confirmed by Figure 9. Figure 9 plots the minimum mean sojourn time $W^{*}$ versus the arrival probability λ when m=3. As explained above, the minimum sojourn time increases as the arrival probability λ increases to $\frac{2m}{m+2+\sqrt{m^{2}+4m}}$ and then decreases with the increase of λ. Figure 10 shows the minimum mean sojourn time $W^{*}$ versus the arrival probability λ for different m=2,3,4,5. It is worth noting that the minimum mean sojourn time decreases as *m* increases when the Type II node is in the energy-limited phase. This is expected because the larger the energy cost of transmission *m*, the more energy is necessary for a data packet transmission, thus the possibility for the Type II node to transmit is likely to decrease and this in turn reduces the possibility of collision, which results in small mean sojourn time. We again see that in Figure 9 and Figure 10 the analytical results match well with the simulation results within reasonable accuracy.

## 6. Conclusions

We considered a slotted-Aloha-based RF energy-harvesting network comprising two nodes. The Type I node has unlimited energy supply and the Type II node is powered by RF energy harvesting where the energy-harvesting opportunities are the RF transmissions by the Type I node. In such a scenario that the two nodes are affected by each other, we set a question of what the optimal transmission probabilities are to minimize the mean packet delay while maximizing the system throughput, subject to the stability condition of the data queue of the Type I node. To get the answer to that question, we proposed an approximate modeling technique to analyze the coupled queueing system. Such an approach provided a closed-form solution for the stationary distribution of the Markov chain, and as a result the optimal transmission probabilities could be obtained in closed-form. We also showed that the Type II node can be in either the energy-limited phase or the energy-unlimited phase by controlling its transmission probability. We showed that for the optimal design of the system, if the data arrival probability of the Type I node is relatively small, the Type II node should be in the energy-limited phase and otherwise the Type II node must be in the energy-unlimited phase by choosing the optimal transmission probability uniquely determined by the arrival probability λ. The accuracy of the proposed approximate model was validated by extensive simulations.
